# Transactions of the College of Physicians of Philadelphia

**Published:** 1876-11

**Authors:** 


### Books Reviewed.

---

*Transactions of the College of Physicians, of Philadelphia.* Third Series, Vol. II. Philadelphia: Lindsay & Blakiston, 1876.

This volume contains the papers read before the College from October, 1875, to July, 1876, inclusive. The contents consist of thirteen papers and a memoir of Dr. George W. Norris and of Dr. John S. Parry. The papers are all of a high character and of great interest. Dr. James H. Hutchiuson reports a case of empyema which, after repeated use of the aspirator, was cured by the introduction of a drainage tube into the chest.

A paper by Dr. Ashhurst, on excision of the knees, is of much value and presents some very important points in this operation. He believes that while diseases of the joints are unquestionably local, that they are not of exclusive local origin, and that general treatment should be combined with the local. He uses a transverse in preference to a **H** or **U** incision. All the papers presented in this volume are of great practical value, and do credit to the high standing of the College.

The volume is presented in a style to correspond with the first of this series, and is finely printed and bound. One chromo-lithograph and several wood cuts are introduced.

